# Influence of Older Age and Other Risk Factors on Pneumonia Hospitalization in Switzerland in the Pneumococcal Vaccine Era

**DOI:** 10.3389/fmed.2019.00286

**Published:** 2019-12-05

**Authors:** Werner C. Albrich, Frank Rassouli, Frederike Waldeck, Christoph Berger, Florent Baty

**Affiliations:** ^1^Division Infectious Diseases and Hospital Epidemiology, Cantonal Hospital St. Gallen, St. Gallen, Switzerland; ^2^Department of Pulmonary and Sleep Medicine, Cantonal Hospital St. Gallen, St. Gallen, Switzerland; ^3^Division of Infectious Diseases and Hospital Epidemiology, University Children's Hospital Zürich, Zurich, Switzerland

**Keywords:** pneumonia, pneumococcus, age, vaccine, indication, incidence, hospitalization

## Abstract

**Background:** Pneumococcal pneumonia is a disease of the extremes of age. However, as other traditional risk factors for pneumococcal pneumonia also increase with older age, it is unclear if older age itself should be an indication for pneumococcal vaccination. Therefore, we assessed the effect of age on risk for hospitalization for pneumonia and for pneumococcal pneumonia.

**Methods:** Using a national hospitalization dataset, all patients ≥16 years hospitalized in a Swiss hospital with a diagnosis of pneumonia or pneumococcal pneumonia between 2002 and 2015 were included. Multivariable logistic regression analysis was used to test the association between age (≥50 or ≥65 years) and hospitalization for pneumonia or pneumococcal pneumonia after adjusting for pneumococcal vaccine indications. Similar analyses were performed for effect of age on length of stay (LOS) and mortality.

**Results:** Among a total of 17,619,016 hospitalizations a diagnosis of pneumonia was present in 421,760 (2.4%) and a diagnosis of pneumococcal pneumonia in 21,610 (0.12%). Age ≥50 years (OR: 3.52 and 2.12, respectively; *p* for both <0.001) and age ≥65 years (OR: 2.98 and 1.80, respectively; *p* for both <0.001) as well as most Swiss pneumococcal vaccine indications were independent predictors of hospitalization with a pneumonia and pneumococcal pneumonia diagnosis, respectively. Older age with both age cut-offs were associated with increased LOS (≥50 years: aRR: 1.19 and 1.24, respectively; age ≥65 years: aRR: 1.60 and 1.20, respectively; *p* < 0.001 for all) and mortality (≥50 years: aOR: 4.73 and 2.84, respectively; age ≥65 years: aOR: 2.38 and 2.69, respectively, *p* < 0.001 for all) in patients with a pneumonia and pneumococcal pneumonia diagnosis, respectively. The effects of pneumococcal vaccine indications decreased with older age. The incidences of hospitalizations with a pneumonia diagnosis and a pneumococcal pneumonia diagnosis increased significantly from the pre-vaccine era to the PCV7 era and the PCV13 era (*p* for trend for both analyses <0.001).

**Conclusion:** This study confirms the Swiss indications for pneumococcal vaccination as independent risk factors for pneumonia hospitalizations. Older age itself should be considered as an additional vaccine indication. Pneumonia and pneumococcal pneumonia in adults have increased despite pneumococcal vaccination in children.

## Introduction

Lower respiratory tract infections and particularly pneumonia as its most severe manifestation, are the leading infectious disease related causes of death worldwide (1). *Streptococcus pneumoniae* is the most common etiology of community-acquired pneumonia (CAP) likely responsible for 40–50% of pneumonias (2–4). The age-related incidence of pneumococcal disease shows a U-shaped curve with highest risks in young children and the elderly (5). Older age itself has been shown to be an independent risk factor for pneumonia (6). It was shown that non-immunocompromising conditions which predispose to pneumococcal disease, such as diabetes mellitus, chronic heart and lung disease, increase with age in elderly adults, and thus play an increasing role as a pneumonia risk factor (7, 8). Furthermore, the presence of these non-immunocompromising conditions, especially if occurring concomitantly, conferred very high risks.

National recommendations for pneumococcal vaccination in adults differ on whether age alone in the absence of other known risk factors, should be a vaccine indication and what the age cut-off should be (9). In Switzerland, age was removed from the indication list for pneumococcal vaccination for adults in 2014 when the 13-valent pneumococcal conjugate vaccine (PCV13) was first recommended and replaced the 23-valent polysaccharide vaccine for persons with any pneumococcal risk factor >5 years of age.

Contemporary data on the incidence of pneumococcal pneumonia in Switzerland are unavailable. However, beside national data on IPD, all hospitalizations with associated ICD-codes as provided by the hospitals are recorded in a national database. In the absence of individually matched microbiology data, *S. pneumoniae* can be assumed the leading etiology among hospitalized patients with an International Classification of Disease version 10 (ICD-10) code for pneumonia.

Using this national hospitalization database we therefore attempted to assess the effect of age as an independent risk factor for hospitalization for pneumonia and for pneumococcal pneumonia, which is vaccine preventable. We also analyzed the effect of age on length of stay (LOS) and mortality in these patients as markers of severity.

## Methods

### Datasets

The hospitalization dataset (https://www.bfs.admin.ch/bfs/de/home/statistiken/gesundheit/erhebungen/ms.html) used in this analysis was provided by the Swiss Federal Office for Statistics. It includes medical information about all hospitalizations in Switzerland since 1998. In this database, the patient information is fully anonymized. No written informed consent was given by patients who were unidentifiable due to the anonymization. The data belong to the Swiss Federal Office for Statistics (Bundesamt für Statistik, Neuchâtel, Switzerland) which provides regulated access to the data for research purpose. The database included geographical and temporal information [patient's age, area of residence, canton of institution, year and month of hospitalization, LOS and discharge information (including death)]. Unique anonymized institution numbers were also available.

The patients' list of diagnoses included one main diagnosis and up to 50 additional diagnoses coded using ICD-10 codes. The datasets used for the demographic data are publicly available and were provided by the Federal Office for Statistics (Statistik des jährlichen Bevölkerungsstandes, ESPOP, for 2002–2010 and Statistik der Bevölkerung und Haushalte, STATPOP, for 2011–2015).

All patients aged ≥16 years who were hospitalized in a Swiss hospital with a pneumonia diagnosis (ICD-10 codes: J13 [pneumonia due to *S. pneumoniae*], J15 [bacterial pneumonia not elsewhere classified], J18 [pneumonia, organism unspecified] listed as either primary diagnosis or non-primary diagnosis) between 2002 and 2015 were included in this analysis. In addition, separate analyses were performed restricted to patients with pneumococcal pneumonia (J13). There were no exclusion criteria.

As pneumonia risk factors, ICD-10 codes were used which represented as closely as possible the conditions and comorbidities which are listed as indications for pneumococcal vaccination according to Swiss recommendations (see Appendix) (10).

In concordance with previous publications (11), the time period from 2002 to 2006 was considered the “pre-vaccine era.” Despite an official recommendation for PCV7 in children <2 years starting in November 2005, the coverage with the 1st dose of PCV7 was only 2% between 2005 and 2007 according to the Swiss National Vaccination Coverage Survey (Durchimpfung bei 2-jährigen Kindern in der Schweiz, Erhebungsperioden 1999–2003, 2005–2007, 2008–2010; https://www.bag.admin.ch/dam/bag/de/dokumente/mt/i-und-b/durchimpfung/tabelle-durchimpfung.xlsx.download.xlsx/tabelle-durchimpfung-de.xlsx). 2007–2010 was considered the “PCV7 era,” when PCV7 was recommended for children <2 years; and 2011–2015 was considered the “PCV13 era” when PCV13 was recommended for children <5 years.

### Statistical Considerations

Proportions of hospitalization due to pneumonia were calculated among persons with and without the respective risk factors. Differences between proportions were tested using χ^2^ tests. Univariate and bivariate (controlling for individual vaccine indications) analyses were performed to assess the effect of age (≥50 years or ≥65 years) and other risk factors on the risk of hospitalization for pneumonia and for pneumococcal pneumonia. Multivariable logistic regression analysis was used to test the association between pneumonia or pneumococcal pneumonia and age (≥50 years or ≥65 years) after adjusting for the presence of risk factors (i.e., individual pneumococcal vaccine indications). Interaction between age and risk factors was calculated to assess for effect modification. As sensitivity analyses, we calculated trends over time separately for pneumonia and pneumococcal pneumonia listed as any diagnosis and as primary diagnosis. Results are reported using odds ratios for hospitalization and in-hospital mortality or relative risks for LOS with 95% confidence intervals. Persons without the respective risk factor were considered the reference group. The associations between continuous variable and the presence of pneumonia or pneumococcal pneumonia diagnosis or risk factors were tested using the Wilcoxon rank sum test. Changes over time were assessed using *X*^2^-test for trend in proportions and Poisson regression. All analyses were done using the R statistical software.

## Results

This study period included a total of 17,619,016 hospitalization cases among which 421,760 cases (2.4%) included a pneumonia diagnosis and 21,610 (0.12%) a diagnosis of pneumococcal pneumonia.

### Risk of Pneumonia Hospitalization

In univariate analyses, age ≥50 years (vs. <50 years) [odds ratio (OR): 4.98, 95%CI: 4.93–5.03] and age ≥65 years (vs. <65 years) (OR: 4.14; 95%CI: 4.11–4.17) were significant risk factors for hospitalization with a diagnosis of pneumonia vs. hospitalization without a diagnosis of pneumonia (Table 1A). All Swiss pneumococcal vaccine indications (based on respective ICD-10 codes) were significantly associated with an increased risk for hospitalization with a diagnosis of pneumonia vs. hospitalization without a diagnosis of pneumonia. Heart failure, chronic lung disease and immunodeficiency conferred the highest risks (Table 1A).

**Table 1A:** Hospitalizations with pneumonia in persons with and without risk factors.

**Risk factor**	**Proportion of hospitalizations** **with pneumonia of all hospitalizations in**		**Univariate analysis**		**Bivariate (controlling for age ≥50, interaction) analysis**			**Bivariate (controlling for age ≥65, interaction) analysis**			**Multivariable analysis with age ≥50**		**Multivariable analysis with age ≥65**	
	**Patients with risk factor**	**Patients** **without risk factor**	**OR**	***p***	**OR RF**	**OR age ≥50**	**Inter-action**	**OR RF**	**OR age ≥65**	**Inter-action**	**OR**	***p***	**OR**	***p***
Age ≥50y	375,591/11,042,751 (3.4%)	46,169/65,76265 (0.7%)	4.98	<0.001							3.52	<0.001		
Age ≥65y	308,868/7,151,622 (4.3%)	112,892/10,467,394 (1.1%)	4.14	<0.001									2.98	<0.001
Heart failure	71,159/597,777 (11.9%)	350,601/17,021,239 (2.0%)	6.43	<0.001	10.0^*^	4.4^*^	0.5^*^	7.6^*^	3.7^*^	0.5^*^	3.16	<0.001	2.94	<0.001
Lung disease (COPD, severe asthma, bronchiectasis)	82,373/758,999 (10.9%)	339,387/16,860,017 (2.0%)	5.93	<0.001	7.8^*^	4.5^*^	0.6^*^	8.3^*^	4.1^*^	0.5^*^	3.73	<0.001	3.71	<0.001
Liver cirrhosis	5,215/110,922 (4.7%)	416,545/17,508,094 (2.4%)	2.02	<0.001	7.0^*^	5.0^*^	0.2^*^	4.3^*^	4.2^*^	0.3^*^	1.25	<0.001	1.47	<0.001
Asplenia	29/682 (4.3%)	421,731/17,618,334 (2.4%)	1.81	0.002	6.8^*^	5.0^*^	0.2^*^	4.3^*^	4.1^*^	0.2^*^	1.41	0.09	1.53	0.03
Chronic kidney disease (nephrotic syndrome)	63,150/713,569 (8.8%)	358,610/16,905,447 (2.1%)	4.5	<0.001	7.6^*^	4.5^*^	0.4^*^	5.9^*^	3.8^*^	0.4^*^	1.96	<0.001	1.77	<0.001
Sickle cell disease	213/2,844 (7.5%)	421,547/17,616,172 (2.4%)	3.3	<0.001	11.7^*^	5.0^*^	0.2^*^	7.5^*^	4.1^*^	0.2^*^	7.80	<0.001	6.45	<0.001
Diabetes mellitus, poorly controlled with heart or renal insufficiency	68,308/124,6291 (5.5%)	353,452/16,372,725 (2.2%)	2.6	<0.001	4.3^*^	4.8^*^	0.4^*^	3.6^*^	4.2^*^	0.5^*^	1.39	<0.001	1.39	<0.001
Lymphoma, leukemia, myeloma	17,134/213,344 (8.0%)	404,626/17,405,672 (2.3%)	3.7	<0.001	5.8^*^	5.0^*^	0.5^*^	5.3^*^	4.2^*^	0.5^*^	2.73	<0.001	2.77	<0.001
CSF leak	246/9,235 (2.7%)	421,514/17,609,781 (2.4%)	1.2	0.10	2.8^*^	5.0^*^	0.3^*^	2.2^*^	4.1^*^	0.3^*^	1.13	0.06	1.21	0.003
Autoimmune disease	12,891/297,036 (4.3%)	408,869/17,321,980 (2.4%)	1.9	<0.001	2.2^*^	5.0^*^	0.7^*^	2.2^*^	4.2^*^	0.7^*^	1.44	<0.001	1.44	<0.001
HIV	1,518/19,428 (7.8%)	420,242/17,599,588 (2.4%)	3.5	<0.001	11.8^*^	5.1^*^	0.2^*^	7.8^*^	4.2^*^	0.3^*^	4.94	<0.001	5.27	<0.001
Immunodeficiency	3,511/26975 (13.0%)	418,249/17,592,041 (2.4%)	6.1	<0.001	12.4^*^	5.0^*^	0.4^*^	10.3^*^	4.2^*^	0.4^*^	3.34	<0.001	3.74	<0.001
Any risk factor (except for age)	223,445/3,110,140 (7.2%)	198,315/14,508,876 (1.4%)	5.6	<0.001	6.5^*^	3.6^*^	0.6^*^	6.3^*^	3.6^*^	0.6^*^				

* *p < 0.001*.

In bivariate analyses and in multivariable logistic regression each vaccine indication and the age categories ≥50 years and ≥65 years were significant independent risk factors for hospitalization with pneumonia (Table 1A). In all bivariate analyses with the outcome pneumonia hospitalization, the interaction term of risk factor^*^age was negatively correlated with the risk of pneumonia hospitalization (OR < 1) indicating a decreasing effect of each risk factor with increasing age.

### Risk of Pneumococcal Pneumonia Hospitalization

When restricted to pneumococcal pneumonia, age ≥50 years (vs. <50 years) (OR: 2.90, 95%CI: 2.80–3.00) and age ≥65 years (vs. <65 years) (OR: 2.42; 95%CI: 2.36–2.49) were significant risk factors for hospitalization with a diagnosis of pneumococcal pneumonia vs. hospitalization without a diagnosis of pneumococcal pneumonia.

All pneumococcal vaccine indications (based on respective ICD-10 codes) except for asplenia were significantly associated with an increased risk for hospitalization with a diagnosis of pneumococcal pneumonia vs. hospitalization without a diagnosis of pneumococcal pneumonia. Chronic lung disease, HIV and immunodeficiency conferred the highest risks (Table 1B).

**Table 1B:** Hospitalizations with pneumococcal pneumonia in persons with and without risk factors.

**Risk factor**	**Proportion of hospitalizations** **with pneumonia of all hospitalizations in**		**Univariate analysis**		**Bivariate (controlling for age ≥50, interaction) analysis**			**Bivariate (controlling for age ≥65, interaction) analysis**			**Multivariable analysis with age ≥50**		**Multivariable analysis with age ≥65**	
	**Patients with risk factor**	**Patients** **without risk factor**	**OR**	***p***	**OR RF**	**OR age ≥50**	**Inter-action**	**OR RF**	**OR age ≥65**	**Inter-action**	**OR**	***p***	**OR**	***p***
Age ≥50y	17,928/11,042,751 (0.2%)	3,682/6,576,265 (0.056%)	2.90	<0.001							2.12	<0.001		
Age ≥65y	13,466/7,151,622 (0.2%)	8,144/10,467,394 (0.1%)	2.42	<0.001									1.8	<0.001
Heart failure	2,566/597,777 (0.4%)	19,044/17,021,239 (0.1%)	3.85	<0.001	6.6^*^	2.7^*^	0.4^*^	4.8^*^	2.2^*^	0.6^*^	1.9	<0.001	2.94	<0.001
Lung disease (COPD, severe asthma, bronchiectasis)	5,196/758,999 (0.7%)	16,414/16,860,017 (0.1%)	7.07	<0.001	8.8^*^	2.5^*^	0.6^*^	9.1^*^	2.3^*^	0.6^*^	5.1	<0.001	5.1	<0.001
Liver cirrhosis	415/110,922 (0.4%)	21,195/17,508,094 (0.1%)	3.10	<0.001	10.4^*^	2.9^*^	0.2^*^	5.7^*^	2.5^*^	0.3^*^	2.0	<0.001	2.2	<0.001
Asplenia	3/682 (0.4%)	21,607/17,618,334 (0.1%)	3.60	0.027	16.5^*^	2.9^*^	0.0	7.9^*^	2.4^*^	0.0	2.1	0.19	2.2	0.17
Chronic kidney disease (nephrotic syndrome)	2,524/713,569 (0.4%)	19,086/16,905,447 (0.1%)	3.14	<0.001	6.0^*^	2.7^*^	0.4^*^	4.6^*^	2.3^*^	0.5^*^	1.5	<0.001	1.5	<0.001
Sickle cell disease	6/2,844 (0.2%)	21,604/17,616,172 (0.1%)	1.72	0.184	3.5^**^	2.9^*^	0.6	2.6^**^	2.4^*^	0.2^*^	2.5	0.02	2.2	0.058
Diabetes mellitus, poorly controlled with heart or renal insufficiency	3,125/1246291 (0.3%)	18,485/16,372,725 (0.1%)	2.22	<0.001	3.1^*^	2.8^*^	0.5^*^	2.9^*^	2.4^*^	0.5^*^	1.3	<0.001	1.3	<0.001
Lymphoma, leukemia, myeloma	1,007/213,344 (0.5%)	20,603/17,405,672 (0.1%)	4.00	<0.001	4.5^*^	2.9^*^	0.7^**^	4.8^*^	2.4^*^	0.6^*^	2.9	<0.001	3.0	<0.001
CSF leak	18/9,235 (0.2%)	21,592/17,609,781 (0.1%)	1.59	0.049	4.8^*^	2.9^*^	0.2^*^	3.4^*^	2.4^*^	0.1^**^	1.6	0.049	1.7	0.03
Autoimmune disease	563/297,036 (0.2%)	21,047/17,321,980 (0.1%)	1.56	<0.001	1.9^*^	2.9^*^	0.7^**^	1.8^*^	2.4^*^	0.7^*^	1.2	<0.001	1.2	<0.001
HIV	246/19,428 (1.3%)	21,364/17,599,588 (0.1%)	10.55	<0.001	2.5^*^	3.0^*^	2.7^*^	17.7^*^	2.5^*^	0.2^*^	11.2	<0.001	11.1	<0.001
Immunodeficiency	255/26,975 (0.9%)	21,355/17,592,041 (0.1%)	7.85	<0.001	14.0^*^	2.9^*^	0.5^*^	11.4^*^	2.4^*^	0.5^*^	3.9	<0.001	4.1	<0.001
Any risk factor (except for age)	11,319/3110140 (0.4%)	10,291/14,508,876 (0.1%)	2.33	<0.001	3.7^*^	2.4^*^	0.6^*^	6.3^*^	2.0^*^	0.6^*^				

* p < 0.001;

** *p < 0.05*.

*Reference groups: persons without the respective risk factor. For multivariable analysis, all risk factors (heart failure, lung disease, liver cirrhosis, asplenia, chronic kidney disease, sickle cell disease, diabetes, lymphoma, leukemia, myeloma, CSF leak, autoimmune disease, HIV, immunodeficiency) were included in 2 analyses, i.e., one analysis for adults ≥50 years and one analysis for adults ≥65 years*.

In bivariate analyses and in multivariable logistic regression each vaccine indication and the age categories ≥50 years and ≥65 years were significant independent risk factors for hospitalization with pneumococcal pneumonia (Table 1B). In all bivariate analyses (except for asplenia) with the outcome pneumococcal pneumonia hospitalization, the interaction term of risk factor^*^age was negatively correlated with the risk of pneumococcal pneumonia hospitalization (OR < 1) indicating a decreasing effect of each risk factor with increasing age.

### Incidence of Pneumonia Hospitalizations Over Time

**Figure 1:**
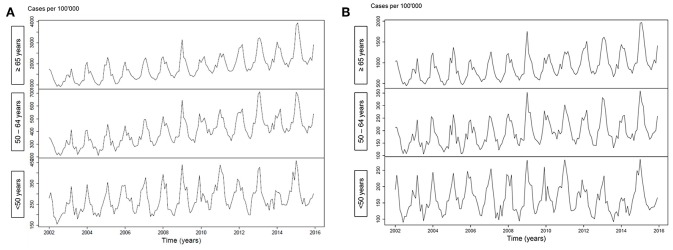
**(A)** Incidence of pneumonia hospitalization (any diagnosis) over time, stratified for age groups. **(B)** Incidence of pneumonia hospitalization (primary diagnosis) over time, stratified for age groups.

**Figure 2:**
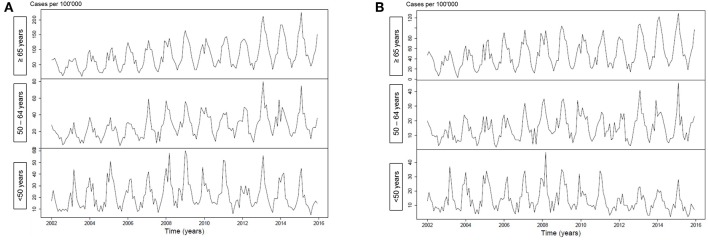
**(A)** Incidence of pneumococcal pneumonia hospitalization (any diagnosis) over time, stratified for age groups. **(B)** Incidence of pneumococcal pneumonia hospitalization (primary diagnosis) over time, stratified for age groups.

**Figure 3:**
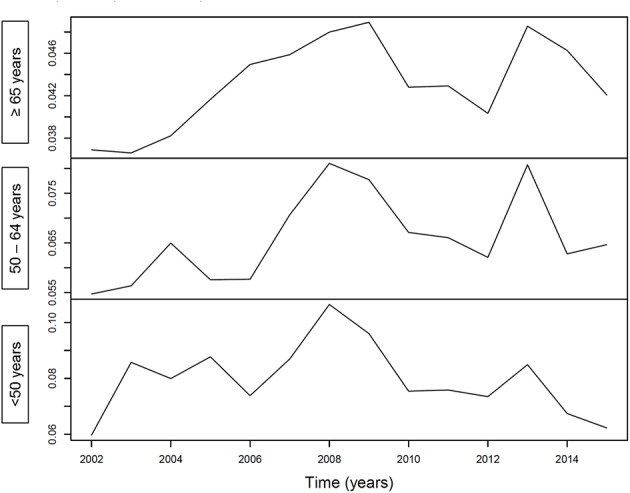
Proportion of pneumococcal pneumonia hospitalizations over time.

Figure 1 illustrates an increasing incidence over time of hospitalization with pneumonia as any diagnosis (Figure 1A) and as primary diagnosis (Figure 1B) over time, stratified for age <50 years (*p* < 0.001 and *p* = 0.64, respectively), age 50–64 years (*p* < 0.001 for both analyses) and for age ≥65 years (*p* < 0.001 for both analyses). For pneumococcal pneumonia hospitalization, there was an increasing incidence as any diagnosis (Figure 2A) or as primary diagnosis (Figure 2B) over time only for age 50–64 years (*p* = 0.001 and *p* = 0.01, respectively) and for age ≥65 years (*p* < 0.001 for both analyses) whereas no increase for age <50 years (*p* = 0.38 and *p* = 0.17, respectively). The proportion of pneumococcal pneumonia showed no clear trend over time as illustrated in Figure 3.

**Figure 4:**
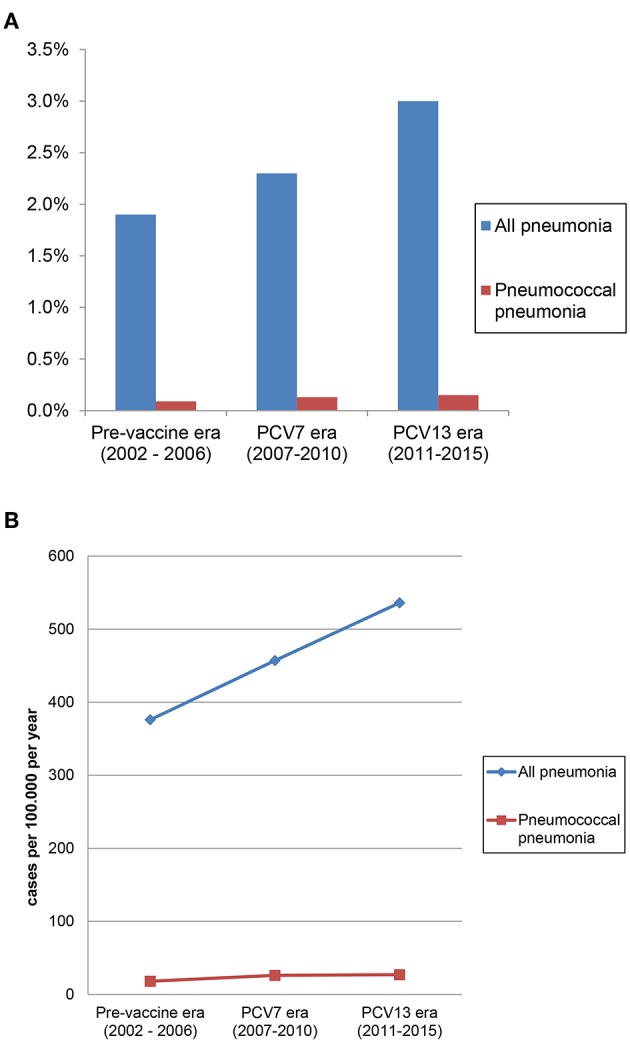
**(A)** Proportion of hospitalizations with pneumonia and pneumococcal pneumonia (among all hospitalizations). **(B)** Incidence of hospitalizations with pneumonia and pneumococcal pneumonia.

The unadjusted proportions of hospitalizations with a pneumonia diagnosis and a pneumococcal pneumonia diagnosis increased significantly from 1.9% in the pre-vaccine era to 2.3% in the PCV7 era and to 3.0% in the PCV13 era, and from 0.09 to 0.13% and 0.15%, respectively (*p* for trend for both analyses <0.001; Table 2; Figure 4). Similarly, the incidences of hospitalizations with a pneumonia diagnosis and a pneumococcal pneumonia diagnosis increased significantly from 376/100,000 inhabitants in the pre-vaccine era to 457/100,000 inhabitants in the PCV7 era and 536/100,000 inhabitants in the PCV13 era and from 18/100,000 to 26/100,000 and 27/100,000, respectively (*p* for trend for both analyses <0.001; Table 2).

**Table 2:** Hospitalizations with pneumonia and pneumococcal pneumonia over time.

	**Pre-vaccine era** **(2002-2006)**	**PCV7 era** **(2007-2010)**	**PCV13 era** **(2011–2015)**	***P* for trend**
Proportions of hospitalizations with pneumonia (among all hospitalizations)	1.9%	2.3%	3.0%	<0.001
Incidence of hospitalizations with pneumonia (/100'000/y)	376	457	536	<0.001
Proportions of hospitalizations with pneumococcal pneumonia (among all hospitalizations)	0.09%	0.13%	0.15%	<0.001
Incidence of hospitalizations with pneumococcal pneumonia (/100'000/y)	18	26	27	<0.001

*The incidence of hospitalizations with pneumonia was calculated per 100,000 inhabitants of Switzerland per year (averaged over the respective era)*.

### Impact of Pneumonia and Pneumococcal Pneumonia Diagnosis on Outcome

In two separate logistic regressions controlling for the presence of any pneumococcal vaccine indication, age ≥50 years (aOR: 4.73; 95%CI: 4.42–5.05) and age ≥65 years (aOR: 2.38; 95%CI: 2.30–2.47) were independent risk factors for in-hospital mortality in patients with a pneumonia diagnosis (Table 3). In two further separate logistic regressions controlling for the presence of any pneumococcal vaccine indication, age ≥50 years [adjusted relative risk (aRR): 1.32; 95%CI: 1.32–1.33] and age ≥65 years (aRR: 1.16, 95%CI: 1.16–1.17) were independent risk factors for increased LOS in hospitalized patients with a pneumonia diagnosis.

**Table 3:** Effect of age and other risk factors on in-hospital mortality and length of stay in patients with a pneumonia diagnosis or a pneumococcal pneumonia diagnosis.

**Risk factor**	**Multivariable analysis in-hospital mortality**			**Multivariable analysis LOS**		
	**aOR**	**95% CI**	***p***	**aRR**	**95% CI**	***p***
**Patients with a pneumonia diagnosis**						
Age ≥50y	4.73	4.12–5.05	<0.001	1.19	1.19–1.19	<0.001
Any risk factor (except for age)	2.32	2.10–2.57	<0.001	1.32	1.32–1.33	<0.001
Interaction	0.49	0.45–0.55	<0.001	0.80	0.79–0.80	<0.001
Age ≥65y	2.38	2.30–2.47	<0.001	1.16	1.16–1.17	<0.001
Any risk factor (except for age)	1.48	1.41–1.55	<0.001	1.24	1.24–1.25	<0.001
Interaction	0.76	0.72–0.80	<0.001	0.83	0.82–0.83	<0.001
**Patients with a pneumococcal pneumonia diagnosis**						
Age ≥50y	2.84	2.22–3.63	<0.001	1.24	1.22–1.25	<0.001
Any risk factor (except for age)	1.89	1.31–2.72	<0.001	1.32	1.29–1.34	<0.001
Interaction	0.60	0.41–0.87	0.008	0.87	0.85–0.89	<0.001
Age ≥65y	2.69	2.25–3.22	<0.001	1.20	1.19–1.21	<0.001
Any risk factor (except for age)	1.69	1.36–2.09	<0.001	1.32	1.30–1.34	<0.001
Interaction	0.60	0.47–0.77	<0.001	0.83	0.82–0.84	<0.001

*LOS, length of stay; aOR, adjusted odds ratio; aRR, adjusted relative risk*.

The interaction terms of any risk factor^*^ age were negatively correlated with mortality and with LOS (OR < 1) indicating a relatively decreasing effect of any risk factor with increasing age.

Similarly, age ≥50 years and age ≥65 years were independent risk factors for in-hospital mortality and for LOS in patients with a pneumococcal pneumonia diagnosis (Table 3). The interaction terms of any risk factor^*^ age were negatively correlated with mortality and with LOS (OR < 1) indicating a relatively decreasing effect of each listed risk factor with increasing age.

## Discussion

This study highlights several relevant aspects of pneumonia etiology in Swiss adults. First, the Swiss indications for pneumococcal vaccine were independent risk factors for hospitalization with both pneumonia and pneumococcal pneumonia. Increasing age was also an independent risk factor for hospitalization with pneumonia and pneumococcal pneumonia after controlling for other known risk factors. Older age was also significantly associated with in-hospital mortality and increased LOS in adults hospitalized with pneumonia and pneumococcal pneumonia. The relative effect of individual risk factors on hospitalization with pneumonia and pneumococcal pneumonia and on markers of severity of pneumonia, i.e., LOS and in-hospital mortality, decreased with older age. And finally, hospitalizations with pneumonia and with pneumococcal pneumonia in persons ≥16 years have increased significantly in Switzerland since 2002.

All indications for pneumococcal vaccination in adults were significantly associated with hospitalization with pneumonia except for CSF leak and asplenia which were not significant or showed non-significant trend. Our data therefore support the current list of age-independent indications for pneumococcal vaccination in adults in Switzerland (10). In addition, older age was also an independent risk factor for hospitalization with pneumonia and for pneumococcal pneumonia. We used age cut-offs of 50 years and of 65 years, which are used as the age cut-offs in the Austrian and US or UK vaccination schedules, respectively. The magnitude of the age effect was similarly high for both age cut-offs (OR 3–3.5 for pneumonia hospitalization and 1.8–2.1 for pneumococcal pneumonia hospitalization) and in the range of some of the best established age-independent risk factors supporting the importance of age. These results are thus in line both with data from other countries with surveillance systems, and international guidelines to recommend pneumococcal vaccination for elderly independent from risk factors. For example, German healthcare claims data for 3.4 million persons during 2009–2012 illustrated that adults ≥60 years without known chronic diseases had similar rates of all-cause pneumonia as adults aged 50–59 years with 1 pneumococcal vaccine indication or adults aged 18–49 years with 2 pneumococcal vaccine indications (12). Similarly, US data from 2006 to 2010 showed that healthy adults ≥65 years had a 4–5 times higher risk of all-cause pneumonia or pneumococcal pneumonia compared to young adults 18–49 years of age (13). Interestingly, in the US study (13), but not in the German study (12), there was also a smaller effect of high-risk conditions in the older age groups confirming the effect modification between age and risk factors in our study. This observation might be explained by the phenomenon of immunosenescence which is also observed in otherwise healthy older persons and therefore ameliorates the difference in pneumonia risk to age-matched persons with chronic conditions.

Our study contributes to a small but increasing number of publications which investigated the effect of pediatric pneumococcal vaccination programs on the incidence of pneumonia in non-vaccinated age groups over time. We noted an increased incidence from the pre-vaccine era to the PCV7 era and to the PCV13 era; increases were most apparent in the age groups 50–64 years and those ≥65 years, This is in line with data from Australia, where no effect on hospitalizations for all-cause pneumonia were detected up to 9 years after introduction of PCV7 into the pediatric vaccination program (14, 15) or the Netherlands up to 3 years after pediatric PCV10 introduction (16). In contrast, Finish (17), Brazilian (18), and some US data indicated a decline in all-cause pneumonia hospitalizations and pneumococcal hospitalizations in at least some non-vaccinated adult age groups (7, 19–21), but usually not in all adult age groups whereas others did not note any declines (22, 23) as recently summarized by Wiese et al. (24). However, the general lack of surveillance data on pneumonia further adds value to our analysis. In addition, our demonstration of similar time trends regardless, whether pneumonia (or pneumococcal pneumonia) was listed as any diagnosis or as primary diagnosis (which likely represent the most severe cases), further strengthens the validity of our results. Of note, our observations extend up to 10 years after PCV7 was recommended for children in Switzerland and 4 years after it was replaced by PCV13 making a lag time bias unlikely as the only explanation for still increasing adult pneumonia hospitalizations.

Limitations of the study include that data were only available for pneumonia cases which required hospitalization. This leads to a selection bias toward more severe pneumonia episodes and reflects the burden of severe pneumonia, but underestimates the total vaccine-preventable burden of pneumonia. Due to the lack of a diagnostic gold standard, pneumonia is a difficult outcome to analyse and can be subject to reporting bias. In addition, this analysis relies on the coding quality of the hospitals. For some PCV13 indications there were no perfectly matching ICD-10 codes. We included pneumonia as the main and the additional diagnosis. Since microbiological diagnoses are neither reliably coded, identified, or even attempted we did not separately analyse episodes of pneumonia due to organisms other than pneumococcus. We also included codes of bacterial pneumonia with other pathogens than pneumococcus given the low sensitivity of pneumococcal etiologic diagnosis (25, 26) and the possibility of undetected coinfection with pneumococcus (27). Our subgroup analysis of only those hospitalizations with pneumococcal pneumonia increased the diagnostic specificity of this vaccine-preventable disease, whereas there is certainly considerably underreporting and therefore decreased sensitivity with this analysis. Thus, our analyses of pneumococcal pneumonia hospitalization incidence are likely an underestimate of the potentially vaccine-preventable pneumococcal disease burden, while the lack of serotype information contributes to this uncertainty. Similarly, predictions of effects of adult pneumococcal vaccination on pneumonia mortality cannot be made based on our data. The presence of collinearity between the independent variables (age and risk factors) cannot be excluded. However, the standard errors associated with our parameter estimates suggest that there is an adequate precision in all our models. The putative presence of collinearity does not seem to affect our findings. Unfortunately, the national hospitalization database does not list age as a continuous variable but only as age categories which does not allow to identify an age cut-off for an increased risk of pneumonia. Nonetheless, our study examines two important age cut-offs, which are used in many national vaccination programs (50 and 65 years).

Important strengths are the comprehensive nationwide coverage and the resulting large number of included pneumonia episodes, which illustrates the magnitude of this public health question. This database represents a unique and important source of information as pneumonia surveillance data are scarce. We confirmed that risk factors which had been selected as indication for pneumococcal vaccination by experts based on previous data or biologic plausibility were indeed associated with risk for pneumonia hospitalization. Our results identify age as an independent risk factor for pneumonia hospitalization and in particular pneumococcal pneumonia hospitalization in Switzerland. Inclusion of age in the list of pneumococcal vaccine indications would increase eligibility for pneumococcal vaccine. This has to be viewed in light of immunosenescence and potentially lower immunogenicity of vaccines in elderly, whereas there was no lower immunogenicity for PCV13 among older age groups in the CAPITA study (28). Other factors to consider are pneumococcal serotype replacement after introduction of pediatric pneumococcal vaccination and unknown effects of future higher-valency vaccines. Importantly, it may facilitate vaccine uptake as annual influenza vaccine is also recommended in Switzerland for all persons ≥65 years and could be performed during the same office visit. Our time trends reflect the impact of pediatric PCV introduction on adult hospitalization as prior to 2014 only the 23-valent polysaccharide vaccine with its limited effect on pneumococcal hospitalization (29) was recommended for persons above age 2 years with pneumococcal risk factors. Future trends in pneumonia hospitalization will have to be monitored to assess the ongoing impact of pediatric PCV implementation.

In conclusion, those conditions which are listed as indications for pneumococcal vaccination by Swiss national vaccination guidelines were confirmed as independent risk factors for pneumonia hospitalizations. Pending cost-effectiveness analyses, older age, even in the absence of additional risk factors, should be considered to be added as an indication for pneumococcal vaccine.

## Data Availability Statement

The datasets generated for this study are available on request to the corresponding author.

## Ethics Statement

Ethical review and approval was not required for the study on human participants in accordance with the local legislation and institutional requirements. Written informed consent from the participants' legal guardian/next of kin was not required to participate in this study in accordance with the national legislation and the institutional requirements.

## Author Contributions

WA and CB had the study idea. WA, CB, and FB developed the study design. WA requested funding and wrote the initial manuscript draft. FB obtained data. WA and FB performed the analysis. WA, FR, CB, and FB interpreted the data. All authors reviewed and commented on the manuscript and agreed with the submitted version.

### Conflict of Interest

WA received an honorarium from GlaxoSmithKline (GSK). His institution has received a honorarium from MSD for a vaccine-independent advisory board. The remaining authors declare that the research was conducted in the absence of any commercial or financial relationships that could be construed as a potential conflict of interest.
