# Supplementary material for: Influence of Older Age and Other Risk Factors on Pneumonia Hospitalization in Switzerland in the Pneumococcal Vaccine Era
Source: Front Med (Lausanne). 2019 Dec 5;6:286. doi: 10.3389/fmed.2019.00286 (PMC6906144; doi:10.3389/fmed.2019.00286)
Supplement: Supplementary file 1 [file Data_Sheet_1.docx]

Appendix:

ICD-10 codes representing conditions qualifying as pneumococcal vaccine indications

|  | Risk | | ICD-10 code | Further characterisation of risk |
| --- | --- | --- | --- | --- |
| **Chronic diseases** | | | |  |
| Heart | | Heart failure | I42.0-9, I25.5, I50.00-01, I50.1, I50.11-4, I50.9 | NYHA ≥ 3 or with deterioration |
| Lung | | Chronic obstructive pneumopathy | At least GOLD 3 or with deterioration  J43, J43.1, J43.2, J43.8, J43.9, J44.- |  |
|  |  | Severe asthma: prolonged or frequent use of oral steroids | J44, J45.- |  |
|  |  | Bronchiectasis from antibody deficiency | J47 |  |
| Liver | | Liver cirrhosis | K70.3, K71.7, K74.-, K74.1, K74.2, K74.3, K74.4, K74.5, K74.6 |  |
| Spleen | | Anatomic or functional asplenia | K73.0, Q89.01 |  |
| Kidney | | Renal insufficiency | P96.0, N14.-, R39.2, D59.3, K76.7, O90.4, N18.-, N18.4, N18.5, N18.8, N18.9 | if creatinine-clearance <30ml/min or with deterioration |
|  |  | Nephrotic syndrome | N04.- |  |
| Blood | | Sickle cell disease | D57.- |  |
| Metabolic | | Poorly controlled Diabetes mellitus with cardiac or renal insufficiency | E10, E11, E12, E13, E14 |  |
| **Neoplasias, transplantation** | | | |  |
| Neoplasias | | Lymphoma, leukemia, myeloma | C81.-, C82.-, C83.-, C84.-, C85.-, C86.-, C88.- C90.-, C91.-, C92.-, C93.-, C94.-, C95.-, C96.-, D61.-, |  |
| Transplantation | | Solid organ transplantation | CHOP: 33.5, 50.5, 37.5, 55.6, 52.8, 46.97, |  |
|  |  | Stem cell transplantation: recipient | CHOP: 41.0B |  |
| **Disorders of the immune system** | | | |  |
| Autoimmunity | | Autoimmune diseases which likely requirre immunosuppression | D59.1, D60.-, D69.3, G35.-, G36.-, G61.0, G61.3, G70.0, K50.-, K51.-, K52.3, K74.3, K75.4, M05.-, M06.1, M07.-, M08., M09.-, M45.0, M08.-, J99.0, M31.4, M31.5, M31.6, M30.2, M30.-, M31.-, M32.-, M33.-, M34.-, M35.-, M36 | prior to start of immunosuppressive therapy |
| Immunosuppression | | Iatrogenic immunosuppression (incl. systemic long-term corticosteroid therapy and radiation therapy) | - |  |
| HIV | | CD4 ≥ 15 %, (adults: ≥ 200 / μl) | B23 |  |
|  |  | CD4 < 15 %, (adults: < 200 / μl) | B21, B22, B24 |  |
| Immuno-deficiency | | Congenital immunodeficiency  Variable immunodeficiency syndrome  Polysaccharide antibody deficiency,  Mannose-binding lectin deficiency | D80.-, D81.-, D82.-, D83.-, D84.-, D89.-, D90 |  |
| **Varia** | | | |  |
| Preterm birth | | delivery <33rd week of pregnancy or birth weight <1500g | - |  |
| ENT | | Cochlea implantat, in situ or planned | - |  |
| Skull | | Base of skull fracture or malformation, cerebrospinal fistula | G96.0, G97.80 |  |
